# Factors affecting the survival probability of becoming a centenarian for those aged 70, based on the human mortality database: income, health expenditure, telephone, and sanitation

**DOI:** 10.1186/1471-2318-14-113

**Published:** 2014-10-21

**Authors:** Jong In Kim, Gukbin Kim

**Affiliations:** Division of Health and Welfare, Wonkwang University, Iksan, 570-749 Republic of Korea; Management with International Business, Royal Holloway, University of London, Egham Surrey, TW20 0EX UK

**Keywords:** Centenarians, Survival probability, Human mortality database, Data quality, Gross national income, Sanitation facilities, Health expenditure, Longevity index, Healthy aging

## Abstract

**Background:**

What are the factors that affect the survival probability of becoming a centenarian for those aged 70? Do the factors include income, health expenditure, the use of mobile telephones, or sanitation? The survival probability of becoming a centenarian (SPBC) is defined as an estimate of the production of centenarians by a population. The SPBC (70) is the survival probability of becoming a centenarian for those aged 70. This study estimates the associations between the SPBC (70), and the gross national income, health expenditure, telecommunications, and sanitation facilities in 32 countries.

**Methods:**

The socioeconomic indicators for this study were obtained from the database of the United Nations Development Programme. In addition, the data for the analysis of centenarians in 32 countries were obtained from the Human Mortality Database, which is maintained by the Department of Demography at the University of California, Berkeley, USA, and the Max Planck Institute for Demographic Research in Rostock, Germany. Associations between socioeconomic indicators and SPBC (70) were assessed using Pearson’s correlation coefficients and multiple regression models.

**Results:**

Significant positive correlations were found between the SPBC (70), and the socioeconomic factors of gross national income (GNI), public expenditure on health as a percentage of gross domestic product (PEHGDP), fixed and mobile telephone subscribers (FMTS) as the standard of living, and improved sanitation facilities (ISF). Overall, the SPBC (70) of female and male predictors were used, in order to form a model production of centenarians, with higher GNI and PEHGDP, as well as higher FMTS and ISF as the socioeconomic factors (R^2^= 0.422, P< 0.001).

**Conclusions:**

The socioeconomic level in all 32 countries appears to have an important latent effect on the production of centenarians in both females and males. This study has identified the following four important aspects of socioeconomic indicators in the survival probability of becoming a centenarian for those aged 70: higher overall economic development level, public expenditure on health, mobile telephone subscribers as the standard of living, and the use of improved sanitation facilities for healthy aging. Thus, the socioeconomic level seems to affect an important on the survival probability of becoming a centenarian.

**Electronic supplementary material:**

The online version of this article (doi:10.1186/1471-2318-14-113) contains supplementary material, which is available to authorized users.

## Background

What are the factors that affect the survival probability of becoming a centenarian for those aged 70? Do the factors include income, health expenditure, the use of mobile telephones, or sanitation?

In many countries, the survival probability of becoming a centenarian (SPBC) has been increasing over the past several decades, particularly among women. While the survival of centenarians is improving, at least in some countries, it is not possible to determine whether the natural life span has increased overall [1]. However, the SPBC may have been affected by the above socioeconomic indicators. This retrospective analysis of the socioeconomic factors, which contribute to the SPBC, may help identify the factors associated with healthy aging.

Today, the population of centenarians is steadily increasing globally [2]. Over the past half-century, the number of centenarians has dramatically increased [3]. The determinants regarding the increase in the number of centenarians include the cohort size at selected ages, along with the probability of survival at selected ages [4]. Namely, the number of centenarians reflects an estimate of the production of centenarians at selected ages, which is the survival probability of becoming a centenarian within a population. Therefore, the SPBC is defined as an estimate of the production of centenarians by a population. Further, the SPBC (70) is the survival probability of becoming a centenarian for those who are aged 70.

Historically, Sachuk [5] first suggested the longevity index in 1970, as the proportion between the number of centenarians and the total population [6]. This value depends on the population structure [7]. The SPBC (70), as an indicator of healthy aging, is based on an unchanged age-specific fertility, as well as on the absence of migration in populations. However, in this study, the SPBC (70), as the centenarian rate has nothing to do with fertility, because the study cohorts have already reached the age of 70. Meanwhile, the probability of survival as the number of centenarians, suggested by Robine and Caselli [4], used to be an indicator of healthy aging in response to the imperfection of the longevity index, thereby resulting in a longevity index that is used in certain countries [4–6, 8, 9].

Healthy aging is an important aspect for the health of centenarians. It requires older people to have access to appropriate improved sanitation facilities; moreover, public expenditure on health and a sense of satisfaction with their level of income, as well as using mobile phones as the standard of living, are essential as well. This paper therefore seeks to determine the association between such socioeconomic indicators, and the SPBC (70).

In particular, although knowledge of the determinants of healthy aging is limited, healthy aging of centenarians is a multifactorial quantitative trait that is influenced by biological, environmental, and psychosocial factors [10]. Among all these elements, socioeconomic factors, as modifiable risk factors, have been researched in several studies. In brief, studies have shown that environmental health factors, such as drinking water and sanitation facilities, can predict mortality in incidences of cancer and diarrhea [11, 12]. However, it is uncertain as to whether these associations are applicable to the SPBC (70), as the production of centenarians. The results concerning the association between the use of telephones, the use of mobile phones, and the improvement of health outcomes, have also been studied [13, 14]. However, it is uncertain as to whether these associations are applicable to the SPBC (70) as the number of centenarians. Finally, previous reports have supported the hypothesis that the associations between national economic indicators and relative fitness among the older population are positive [15]; however, these relations have not been confirmed in other studies of centenarians [16]. Although the socioeconomic indicators, as modifiable risk factors, have been studied in several studies, it is uncertain as to whether these associations are applicable to the production of centenarians.

Therefore, this study has examined the associations of independent socioeconomic indicators with SPBC (70), as the number of centenarians. Further, this study estimates the influence that correlates between the SPBC (70) and gross national income, health expenditure, the use of mobile telephones, and sanitation facilities, as the socioeconomic indicators in 32 countries. Overall, this study aims to estimate the association between the SPBC (70) and socioeconomic factors, by using Pearson’s correlation coefficients and multiple regression models.

## Methods

### Estimation of the SPBC (70)

**Figure 1:**
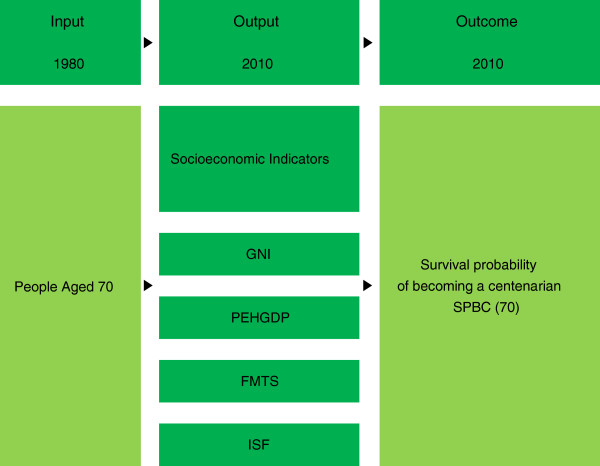
**Description of theoretical framework.**

This study’s objective is to identify the differences in the socioeconomic indicators related to the SPBC (70) within 32 countries. The SPBC (70) has the advantage of controlling other potential confounders, such as infant mortality, which affect the number of centenarians, and overcoming the problem of migration inherent in the change of nationality. The SPBC (70) is the number of survivors who are aged 100 at a given date, divided by the size of the corresponding cohort at a given age [4, 9]. This study utilized the Human Mortality Database (HMD) of centenarians from 32 countries for calculation [17]. The HMD classifies these countries into several categories by the quality of age-reporting data at very old ages. Therefore, we have used data from the human mortality database, where most countries have relatively good data quality to perform their analysis. In order to become a centenarian, it takes a period of over 30 years for those who are aged 70, which is the years of data used in all countries from 1980 to 2010. As a result, with regard to the last thirty years, the SPBC (70) reflects the number of those aged 100 in the year 2010, divided by the size of the corresponding cohort at the age of 70 in 1980 [SPBC (70)= (The number of those aged 100 in 2010/The number of those aged 70 years in 1980)* 10000]. This calculation implies that it is the number of people aged 70 in 1980 who reached 100 in 2010, as a retrospective study, as well as an estimate of the production of centenarians (see Additional file 1). The SPBC (70) comprises cohorts who were born in 1910. The meaning of SPBC (70) is to live until those aged 70 years become centenarians. More specifically, life expectancy is the expected number of years of life remaining at a given age. However, the SPBC (70), which is an indicator for assessing the production of centenarians, or the number of centenarians by a population, reflects the longevity of a population in 32 countries, or the survival probability of becoming centenarians for those aged 70 (Figure 1).

### The framework of the factors that affect survival

The proposed framework of this study depicts the socioeconomic factors for SPBC (70), as the number of survivors who are centenarians (Figure 1). In fact, centenarians have been an influence on the socioeconomic factors for this period.

Healthy aging of centenarians is a multifactorial quantitative trait, which is influenced by biological, psychosocial, and environmental factors [10, 18]. Healthy aging in centenarians indicates the physical activity of living for at least a hundred years with the absence of diseases, alongside being characterized by the preservation of functional capacity and social well being, as exemplified through active life in society [9, 19–21]. In particular, the SPBC (70), as the production of centenarians, may be affected under the control of a social environment and a hereditary factor. However, the SPBC (70) of this study excludes the effect by hereditary factors. The reason as to why the individual-level factors are omitted in the study provides the notion that individual characteristics are closely linked to mortality, because that *ceteris paribus* assumption in individual characteristics, which correlates to SPBC (70), only has to consider the socioeconomic level from a macroscopic viewpoint.

Although the biological factors could be directly impacted by physical factors, they are indirectly affected by environmental health factors. These factors could be found in drinking water and sanitation facilities [11, 12]. Thus, as a higher proportion of the population uses improved sanitation facilities (ISF), there will be a greater promotion of health as a preventive measure against waterborne infections [22, 23] and sanitary-related gastroenteric diseases, such as diarrhea [24], among the older population.

On the other hand, the association between the use of telephones and improved health has also been studied [13, 14]. The hypothesis of this study is that the associations between fixed and mobile telephone subscribers (FMTS), as the standard of living of socioeconomic factors, and the SPBC (70) may differ among centenarians in developed countries, or be comparable with those in undeveloped countries.

Finally, although the associations between health expenditures and their relationship with the number of centenarians in 32 OECD countries [9], along with the national income and healthcare spending in their relationship with mortality among the older population, are correlated [15], it is uncertain as to whether these associations are applicable to the number of centenarians with social well-being, such as public expenditure on health as a percentage of gross domestic product (PEHGDP) and gross national income (GNI), among the study of 32 countries.

Hence the hypothesis, that the associations between SPBC (70) as outcomes, and sanitation facilities (ISF), use of mobile telephones (FMTS), health expenditure (PEHGDP), and national income (GNI) as outputs, can predict the survival probability of becoming centenarians. These factors could be explained by healthy aging, which affects the survival probability of becoming centenarians; the SPBC (70) deals with the survival probability of becoming 100 years in 2010, for those aged 70 in 1980 (Figure 1).

### Deriving the hypothesis, and setting the models

In order to examine the association between the SPBC (70) and the socioeconomic factors of healthy aging, we need to set a study model for each variable. This model was used to estimate the SPBC (70), in terms of the socioeconomic indicators. The models depict the framework proposed herein of the socioeconomic factors of healthy aging, according to the variables selected. The three models yielded the following results. The SPBC (70), as predictors, was used to form a model combination, as the nub of the economic factors GNI and PEHGDP, with FMTS and ISF as social factors. These variables are reflective of the health expenditure and sanitation factors, as well as the economic development level and telecom attainment indicators. Thus, model 1 is the SPBC (70) of females and males, model 2 is the SPBC (70) of males, and model 3 is the SPBC (70) of females. All of the models were used to form a model with GNI and PEHGDP, and FMTS and ISF factors for the production of centenarians, which may differ among the elderly in all 32 countries. Therefore, from this model, we derived a hypothesis stating that increases in GNI and PEHGDP, which are earmarks of income, as well as increases in FMTS and ISF, will lead to a corresponding increase in the SPBC (70) in all 32 countries. In contrast, decreases in GNI and PEHGDP, as well as decreases in FMTS and ISF, will result in a corresponding decrease in the SPBC (70).

### Data collection and estimate of socioeconomic indicators

Ethics approval for the study was obtained from the Institutional Review Board Wonkwang University (WKIRB-201409-SB-057). The data for the analysis of the centenarians were obtained from the Human Mortality Database (HMD), which is maintained by the Department of Demography at the University of California, Berkeley, USA, and the Max Planck Institute for Demographic Research in Rostock, Germany [17]. The countries and overseas island dependencies were selected according to the classification system applied by the United Nations.

For the SPBC (70) analyses, this study excluded countries for which information on the number of centenarians was insufficient. A total of 32 countries were selected. The socioeconomic factors for this study were obtained from the data by country in the United Nations Development Programme [25], and a dataset in the United Nations database [26]. Because the SPBC (70) focuses on the cohorts for over a 30-year period, the socioeconomic factors during the period would have largely changed. Therefore, it is proper to examine the association between the SPBC (70) and socioeconomic indicators. There are at least time series data for using a telephone, economic development level, health expenditure, and sanitation, which will make the results more robust. Further, there are two comparability issues for the present study. One is comparability across countries, whereas the other is comparability across periods from 1980 to 2010, which the socioeconomic factors have considered. Because the SPBC (70) is a type of dynamic and outcome indicator (Figure 1) that represents the improvement of mortality that is closely related to the country-level socioeconomic development, we need to employ the equivalent indicator.

Therefore, the SPBC has been used as the mean rate of change index (MRCI), i.e. the growth rate or rate of decline, in order to measure the change of socioeconomic indicators during the past thirty years. The MRCI was used to supplement faults in such information, because some countries experienced greater progress in 1980 to 2010, whereas some did not. The MRCI can be maintained not only by the present value of the indicator, but also can consider the change rate in 1980 to 2010. The MRCI is the change in the value of a quantity divided by the elapsed time [27, 28]. Further, the socioeconomic indicators in this study reflect the MRCI, the sum of the change in the value of quantity indications from 1980 to 2010, divided by the number (N) of elapsed years [The MRCI= (The present value at that time of indicators in 1980 + The value of change indicators of the growth rate or rate of decline in 2010)/N], as an estimate of the time series data of socioeconomic development indicators. However, this study excluded those indicators for which information on socioeconomic development indicators was insufficient in the time series data.

The following factors were used: *(1) GNI (Gross National Income) per capita (constant 2005 international $)* [25]: aggregate income of an economy generated by its production and ownership of factors of production, less the incomes paid for the use of factors of production owned by the rest of the world, converted to international dollars using purchasing power parity (PPP) rates, divided by midyear population, from 1990 to 2010; *(2) FMTS (Fixed and mobile telephone subscribers per 100 people)* [25]: sum of telephone lines and mobile subscribers, expressed per 100 people, from 1980 to 1990, and from 1980 to 2000; *(3) PEHGDP (Expenditure on health, public% of GDP)* [25]: current and capital spending from government central and local budgets, external borrowings and grants, including donations from international agencies and nongovernmental organizations, and social or compulsory health insurance funds, expressed as a percentage of GDP, from 2000 to 2010; and *(4) ISF (Improved sanitation facilities, urban)* [26]: access to improved sanitation facilities refers to the percentage of the population using improved sanitation facilities. The improved sanitation facilities include flush/pour flush (to piped sewer system, septic tank, pit latrine), ventilated improved pit (VIP) latrine, pit latrine with slab, and composting toilet from 2005 to 2010.

### Descriptive statistics

Table 1 presents the descriptive statistics for this range of SPBC (70), along with the socioeconomic factors. The SPBC (70) MF of the 32 countries ranged from 19 in the Ukraine, to 250 in Japan. The SPBC (70) M of the 32 countries ranged from 9 in the Ukraine, to 83 in Japan. The SPBC (70) F of the 32 countries ranged from 24 in the Ukraine, to 381 in Japan. The mean ISF ranged from 75% in Russia, to 100% in Japan, Canada, Switzerland, France, Australia, and the United Kingdom, with a mean of 97.85%. The mean FMTS ranged from 7.05 in Poland, to 65.85 in Sweden, with a mean of 30.76. The mean PEHGDP ranged from 3.2 in Russia, to 8.65 in France, with a mean of 6.16. Lastly, GNI was the lowest in the Ukraine (GNI, $7,095), and was the highest in Luxembourg (GNI, $44,679), with a mean of $23,889 across all 32 countries.

**Table 1:** **Descriptive statistics of variable**

Variable	N	Mean	StDev a	Minimum	Maximum
SPBC (70) MF	32	80.28	51.36	19	250
SPBC (70) M	32	32.41	19.05	9	83
SPBC (70) F	32	116.7	79.6	24	381
GNI	32	23889	9627	7095	44679
PEHGDP	32	6.16	1.52	3.2	8.65
FMTS	32	30.76	16.03	7.05	65.85
ISF	32	97.85	5.51	75.02	100

a: Standard deviation.

SPBC (70): Survival probability of becoming a centenarian for those aged 70 (per 10,000).

MF: Females and Males, M: Males, F: Females.

GNI: Gross National Income per capita (constant 2005 international $) (1990–2010).

PEHGDP: Public expenditure on health as a percentage of GDP (% of GDP) (2000–2010).

FMTS: Fixed and mobile telephone subscribers (per 100 people) (1980–1990).

ISF: Proportion of the population using improved sanitation facilities (%), urban (2005–2010).

## Results

### The impact and prediction variables of SPBC (70)

**Figure 2:**
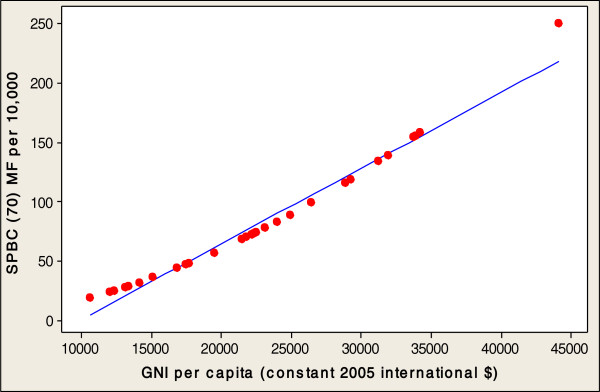
**SPBC (70) MF associated with GNI in 32 countries.**

**Figure 3:**
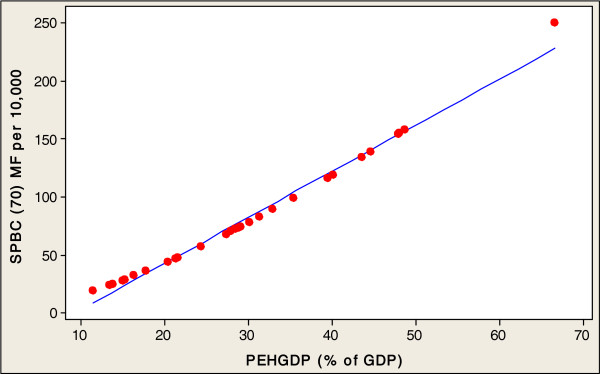
**SPBC (70) MF associated with PEHGDP in 32 countries.**

**Figure 4:**
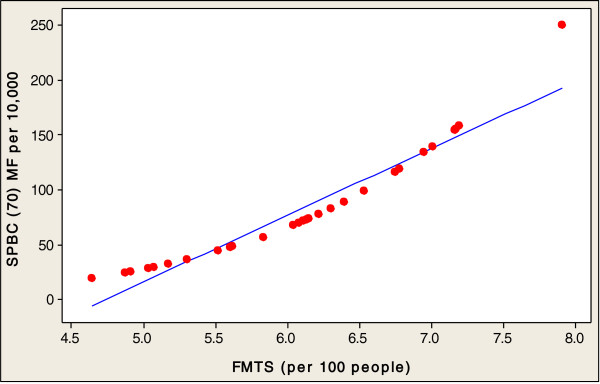
**SPBC (70) MF associated with FMTS in 32 countries.**

**Figure 5:**
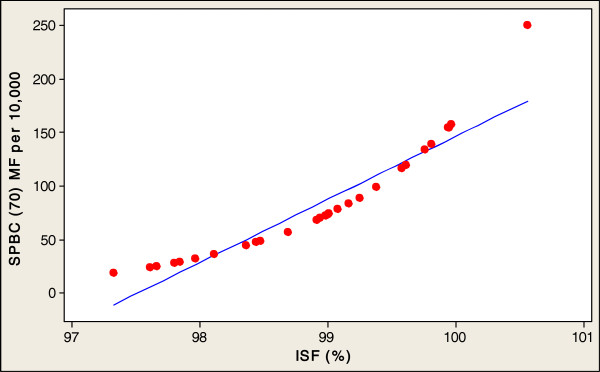
**SPBC (70) MF associated with ISF in 32 countries.**

In order to investigate the direct relationships between the socioeconomic variables involved in healthy aging and SPBC (70) in the 32 countries studied, this paper conducted a multiple regression analysis. Table 2 presents an analysis of the socioeconomic factors related to the SPBC (70) in all 32 countries. The fitted line plots of country characteristics against the SPBC (70) are presented, in order to indicate the strength of the associations between these socioeconomic factors and the production of centenarians. Significant positive correlation coefficients were found between the SPBC (70), and the entire socioeconomic factors of GNI (r=0.555, p<0.001), PEHGDP (r=0.583, p<0.001), ISF (r=0.382, p<0.0311) and FMTS (r=0.611, p<0.001) (Table 2). A log scale was used for all explanatory variables (see Figures 2, 3, 4 and 5). The regression analysis of the socioeconomic factors discovered the prediction of univariate variables for SPBC (70) in 32 countries (Table 3), which are the strongest predictors among the three regression models (Tables 4 and 5). The SPBC (70) of female predictors was used to form a model regarding the production of centenarians, including higher GNI, FMTS, PEHGDP, and ISF factors (R^2^= 0.439, P<0.003); however, SPBC (70) of males predictors was also used to form a model with higher GNI, FMTS, PEHGDP, and ISF factors (R^2^= 0.303, P<0.039). Finally, the SPBC (70) of female and male predictors were used to form a model production of centenarians with higher GNI and FMTS, as well as higher PEHGDP and ISF as socioeconomic factors (R^2^= 0.422, P< 0.004) (see Table 5).

**Table 2:** **Correlations of the indications with SPBC (70)**

P-Value		Correlations coefficient	P-Value
SPBC (70) MF	GNI	0.555	0.0001
	PEHGDP	0.583	0.0001
	FMTS	0.611	0.0001
	ISF	0.382	0.0311
SPBC (70) M	GNI	0.421	0.0017
	PEHGDP	0.457	0.0081
	FMTS	0.521	0.0021
	ISF	0.351	0.0491
SPBC (70) M	GNI	0.568	0.0001
	PEHGDP	0.594	0.0001
	FMTS	0.623	0.0001
	ISF	0.391	0.0281

SPBC (70): Survival probability of becoming a centenarian for those aged 70 (per 10,000).

MF: Females and Males, M: Males, F: Females.

GNI: Gross National Income per capita (constant 2005 international $) (1990–2010).

PEHGDP: Public expenditure on health as a percentage of GDP (% of GDP) (2000–2010).

FMTS: Fixed and mobile telephone subscribers (per 100 people) (1980–1990).

ISF: Proportion of the population using improved sanitation facilities (%), urban (2005–2010).

**Table 3:** **Univariate variables for the SPBC (70)**

	Variable	Coefficient	T-Value	P-Value	R ^2^
SPBC (70) MF	GNI	0.002	3.65	0.001	0.308
	PEHGDP	19.87	3.93	0.001	0.341
	FMTS	1.966	3.93	0.001	0.371
	ISF	3.583	2.26	0.003	0.146
SPBC (70) MF	GNI	0.008	2.54	0.001	0.177
	PEHGDP	5.751	2.82	0.008	0.209
	FMTS	0.619	3.35	0.002	0.272
	ISF	1.212	2.04	0.049	0.122
SPBC (70) MF	GNI	0.004	3.78	0.001	0.322
	PEHGDP	31.19	4.05	0.001	0.353
	FMTS	3.089	4.36	0.001	0.388
	ISF	5.635	2.32	0.028	0.152

SPBC (70): Survival probability of becoming a centenarian for those aged 70 (per 10,000).

MF: Females and Males, M: Males, F: Females.

GNI: Gross National Income per capita (constant 2005 international $) (1990–2010).

PEHGDP: Public expenditure on health as a percentage of GDP (% of GDP) (2000–2010).

FMTS: Fixed and mobile telephone subscribers (per 100 people) (1980–1990).

ISF: Proportion of the population using improved sanitation facilities (%), urban (2005–2010).

**Table 4:** **Multiple regression models for predicting SPBC (70)**

	Variable	Coefficient	T-Value	P-Value	R ^2^
SPBC (70) MF	GNI	0.001	1.28	0.001	0.376
	PEHGDP	13.01	1.78		
	FMTS	1.763	3.93	0.001	0.391
	ISF	1.385	0.92		
	PEHGDP	10.47	1.56	0.001	0.421
	FMTS	1.277	2.01		
SPBC (70) MF	GNI	0.003	0.77	0.005	0.225
	PEHGDP	4.052	1.35		
	FMTS	0.541	2.63	0.007	0.291
	ISF	0.536	0.89		
	PEHGDP	2.311	0.84	0.007	0.289
	FMTS	0.467	1.81		
SPBC (70) MF	GNI	0.002	1.35	0.001	0.392
	PEHGDP	20.23	1.82		
	FMTS	2.769	3.52	0.001	0.406
	ISF	2.181	0.95		
	PEHGDP	16.41	1.61	0.001	0.438
	FMTS	2.011	2.09		

SPBC (70): Survival probability of becoming a centenarian for those aged 70 (per 10,000).

MF: Females and Males, M: Males, F: Females.

GNI: Gross National Income per capita (constant 2005 international $) (1990–2010).

PEHGDP: Public expenditure on health as a percentage of GDP (% of GDP) (2000–2010).

FMTS: Fixed and mobile telephone subscribers (per 100 people) (1980–1990).

ISF: Proportion of the population using improved sanitation facilities (%), urban (2005–2010).

## Discussion

This study investigated the socioeconomic factors associated with the SPBC (70). The present SPBC (70) MF, which is based on a study of centenarians and factors from the national censuses of 32 countries examined in this study, indicated an average of 80.28 centenarians per 10,000. In this study, the SPBC (70) was used to help create a model identifying the socioeconomic factors on the survival probability of becoming a centenarian for those aged 70.

First, *the social factor of ISF* (2005–2010), which indirectly reflects the environmental health [11, 12, 22–24] that is necessary for the survival probability of becoming centenarians, was included in all Models 1, 2 and 3 of females and males, which were found to be a significant factor for the production of centenarians. In the current study, the ISF in Russia (ISF=75%) was the lowest among all 32 countries; whereas, those of Japan, Canada, Switzerland and United Kingdom (ISF=100%) were the highest. The ISF is likely to be a major contributing factor to a high SPBC (70) in certain countries. The mortality rate stemming from the ISF is also a well-known public health concern. This implies that older people who are vulnerable to the risk of ISF might not have the same life expectancy as centenarians. In Russia, the occurrence of typhus epidemic, particularly in circumstances where people are interned under poor hygienic conditions, should be regarded as a barometer for the general social situation. If these indicators are ignored, the consequences could be disastrous [29]. These examples should highlight the fact that immediate intervention to combat the louse epidemic is essential, in order to eradicate emerging typhus among the general population of Russia [29]. This notion means ensuring that the importance of sanitation for infectious diseases is recognised. On the other hand, Russia has the lowest ISF for the production of centenarians, because both of these variables are reflective of a lack of any government investment in health and hygiene facilities infrastructure. The production of centenarians in this study has been indirectly affected by the environment health factors, namely the ISF. Therefore, among the centenarians, the higher the ISF, the greater is the promotion of health as a preventive measure against infectious diseases [22, 23] and sanitary facilities [24]. Simultaneously, using the ISF might help maintain the requisite environmental health for the production of centenarians. Therefore, the predictors’ correlations between the SPBC (70) and ISF are strongly positive, which may well indicate that the ISF is an important independent contributor to the survival probability of becoming centenarians.

**Table 5:** **Multiple regression models for predicting SPBC (70)**

Model 1	
(1) Y_1_ = - 50.959 + 2.450E-5 X_1_ + 9.692 X_2_ + 1.268 X_3_ + 0.327 X_4_ + En	
	R^2^= 0.422, F-Value=4.933, P=0.004
Model 2	
(2) Y_2_ = - 30.612 + 0.00001 X_1_ + 1.984 X_2_ + 0.586 X_3_ + 0.412 X_4_ + En	
	R^2^ = 0.303, F-Value =2.939, P = 0.039
Model 3	
(3) Y_3_ = - 89.981 + 0.00001 X_1_ + 15.034 X_2_ + 1.974 X_3_ + 0.512 X_4_ + En	
	R^2^ = 0.439, F-Value = 5.278, P = 0.0031

Model 1, 2, 3:

: Economic level (+) Health expenditure (+) Using fixed and mobile telephone (+) Sanitation.

Y_1_ = SPBC (70) MF.

Y_2_ = SPBC (70) M.

Y_3_ = SPBC (70) F.

X_1_ = GNI.

X_2_ = PEHGDP.

X_3_ = FMTS.

X_4_ = ISF.

SPBC (70): Survival probability of becoming a centenarian for those aged 70 (per 10,000).

MF: Females and Males, M: Males, F: Females.

GNI: Gross National Income per capita (constant 2005 international $) (1990–2010).

PEHGDP: Public expenditure on health as a percentage of GDP (% of GDP) (2000–2010).

FMTS: Fixed and mobile telephone subscribers (per 100 people) (1980–1990).

ISF: Proportion of the population using improved sanitation facilities (%), urban (2005–2010).

Second, *the social factor of FMTS* (1980–2000), which indirectly reflects the standard of living, can contribute to the health aspect, by accessing health-related information [13, 14], as well as the high-levels in the quality of life [9]; these were included in all Models 1, 2, and 3 of females and males. Increases in FMTS led to an increase in SPBC (70) values, suggesting that they are significant contributory factors to the healthy aging and production of centenarians. In the current study, the FMTS score in Poland (FMTS=7) was the lowest among all 32 countries; whereas, that of Sweden (ISF=66) was the highest. The FMTS is likely to be a major contributing factor to a higher SPBC (70) in 32 countries. Therefore, the FMTS was included in all Models 1, 2 and 3 of females and males, which indirectly reflect the health factors for both social well-being and standard of living, which are necessary for healthy aging and the production of centenarians; further, they were found to be significant factors of healthy aging (Tables 4 and 5). This result implies that the current study has high correlations between FMTS and the survival probability of becoming centenarians, because both of these variables are reflective of the government’s investment in telecommunications infrastructure in health, high-level in the quality of life, and individual income of citizens in developed countries [9]. On the other hand, if an individual’s mental health improves by periodic telephone counseling, then human relationships, which maintain social relationships with family and friends in the oldest-old, might have beneficial effects on mental health [30, 31]. Therefore, periodic telephone counseling appears to have an important latent effect on mortality into late life [31]. Moreover, telemedicine and conventional health services are complementary for enhancing the health status in an aging society [32]. The FMTS is an important independent contributor to the survival probability of becoming centenarians for those aged 70 (Tables 4 and 5).

Finally, *economic factors* of PEHGDP (2000–2010) and GNI (1990–2010), which indirectly reflect the standard of living and income level [9, 15] that are necessary for the survival probability of becoming centenarians, were included in all Models 1, 2 and 3 of females and males; moreover, they were found to be a significant factor in the production of centenarians. In the current study, the PEHGDP in Russia (PEHGDP=3.2) and GNI in the Ukraine (GNI= 7095) were the lowest among all 32 countries; whereas, the PEHGDP in France (PEHGDP=8.65) and GNI in Luxembourg (GNI=44679) were the highest. The PEHGDP and GNI are likely to be major contributing factors to a high SPBC (70) in certain countries. Increases in the GNI and PEHGDP led to an increase in the SPBC (70), thereby suggesting that the PEHGDP and GNI are significant factors of the production of centenarians in all models of females and males. Although the relationship of economic indicators with health is unclear, lower income has been associated with morbidity [15]. In fact, death rates exceeded emergency thresholds at 4 sites during epidemics of *Plasmodium falciparum* malaria in Burundi [33]. However, in higher-income countries, the prevalence of frailty is lower, and people live longer [15]. Therefore, Models 1, 2, and 3 of females and males predictors’ correlations between the SPBC (70) and the PEHGDP and GNI are all strongly positive, which may well indicate that the PEHGDP and GNI are important independent contributors to the production of centenarians, and the survival probability of becoming centenarians.

In the meantime, we have examined whether these factors have the same impact on both females and males [34]. The SPBC (70) of females and males, an indicator for assessing the production of centenarians, reflects the overall economic development level, mobile telephone subscribers, health expenditure, and sanitation facilities level in 32 countries. However, females have more to do with the power of explanation of these factors, than do males (Table 5; Males, R^2^= 0.303; Females, R^2^= 0.439)).

Meanwhile, although the data quality of HMD is better compared to the UN data, the HMD data are not free from biases. For example, age exaggeration at very old ages in the U.S.A., as well as in some other countries, is not uncommon [35]. One of the simplest ways to check this is to compare the percentage distribution from age 95 to age 105 (see Additional file 2, Additional file 3). If a distribution is not in line with those of Sweden or Japan, the data quality of centenarians is likely poor [36]. The results of the data quality assessment of centenarians might have possible biases of such data.

The limitations regarding the accuracy of development variables are insufficient information for some countries over the past thirty years from 1980 to 2010. However, this study has used the mean rate of change index (MRCI), by considering the time and outcome, such as considering the growth rate, or rate of decline, in order to measure the change of socioeconomic indicators during the past thirty years.

Hence, the hypothesis states that the associations between SPBC (70) and socioeconomic factors (GNI, PEHGDP FMTS, ISF) could predict the healthy aging and production of centenarians. Hence, these factors could be explained by the survival probability of becoming centenarians that affects the healthy living of centenarians. For the proposed Models 1, 2, and 3 of females and males, if countries were to have higher GNI and PEHGDP, and higher FMTS and ISF factors, these factors would surely have an impact on the increase of SPBC (70) in all 32 countries.

## Conclusion

The socioeconomic level in all 32 countries appears to have an important latent effect on the production of centenarians in both females and males. This study has identified the following four important aspects of socioeconomic indicators in the survival probability of becoming a centenarian for those aged 70: higher overall economic development level, public expenditure on health of GDP, mobile telephone subscribers as the standard of living, and the use of improved sanitation facilities for healthy aging. Thus, the socioeconomic level seems to affect an important on the survival probability of becoming a centenarian.

## Electronic supplementary material

Additional file 1: **A list as the data assessment of SPBC (70) for study 32 countries.** (PDF 73 KB)

Additional file 2: **The number of distribution age 95 to 105 for selected countries in 2010.** (PDF 58 KB)

Additional file 3: **A percentage distribution age 95 to 105 for selected countries in 2010.** (PDF 58 KB)
