# Supplementary material for: Factors affecting the survival probability of becoming a centenarian for those aged 70, based on the human mortality database: income, health expenditure, telephone, and sanitation
Source: BMC Geriatr. 2014 Oct 21;14:113. doi: 10.1186/1471-2318-14-113 (PMC4216852; doi:10.1186/1471-2318-14-113)
Supplement: Supplementary file 1 — Additional file 1: A list as the data assessment of SPBC (70) for study 32 countries. (PDF 73 KB) [file 12877_2014_1048_MOESM1_ESM.pdf]

[Additional file 1] A list as the data assessment of SPBC (70) for study 32 countries

Countries	2010 (A) †			1980 (B) ‡			SPBC (70) ±		
	Female	Male	Total	Female	Male	Total	Female	Male	Total
Japan	14631	2473	17103	383540	299372	682912	381	83	250
Canada	1805	372	2177	74706	62642	137348	242	59	158
Iceland	15	5	20	665	627	1292	226	80	155
France	5959	934	6893	257715	190525	448240	231	49	154
Australia	968	230	1198	47118	38903	86021	205	59	139
U.S.A	16403	3290	19693	843024	631777	1474801	195	52	134
Spain	2474	614	3088	147872	111746	259618	167	55	119
Italy	4661	968	5629	273638	213501	487139	170	45	116
Switzerland	433	94	528	30419	22911	53330	142	41	99
United Kingdom	3949	691	4640	297202	226439	523640	133	31	89
Sweden	582	118	700	45268	38897	84165	129	30	83
Denmark	301	59	360	25350	20692	46042	119	29	78
Luxembourg	15	3	18	1342	966	2308	114	28	78
Netherlands	632	98	730	56049	42468	98517	113	23	74
Portugal	427	96	522	40076	30398	70474	106	31	74
Belgium	548	79	626	49186	36685	85871	111	21	73
Austria	455	76	531	45534	28576	74110	100	27	72
Germany	5015	684	5700	495622	298265	793887	101	23	72
Norway	213	43	256	20015	16354	36369	106	26	70
Finland	223	41	264	23934	15034	38968	93	27	68
Ireland	113	20	133	12181	11115	23297	92	18	57
Poland	919	243	1163	140938	101952	242890	65	24	48
Lithuania	76	30	106	14477	8182	22659	53	37	47
Estonia	39	11	50	7550	3830	11381	52	29	44
Belarus	194	38	232	44364	20912	65276	44	18	36
Hungary	249	67	316	56485	41433	97918	44	16	32
Latvia	49	8	58	12764	6884	19648	39	12	29
Slovakia	79	25	104	20587	16369	36956	39	15	28
Bulgaria	117	54	170	35729	31835	67564	33	17	25
Czech Republic	195	47	241	55505	40013	95517	35	12	25
Russia	1975	291	2266	661607	265041	926648	30	11	24
Ukraine	641	109	749	264466	123511	387977	24	9	19

†: The number of aged 100 in 2010

‡: The number of people aged 70 years in 1980

±: (A) / (B) *10000
