# Supplementary material for: Factors affecting the survival probability of becoming a centenarian for those aged 70, based on the human mortality database: income, health expenditure, telephone, and sanitation
Source: BMC Geriatr. 2014 Oct 21;14:113. doi: 10.1186/1471-2318-14-113 (PMC4216852; doi:10.1186/1471-2318-14-113)
Supplement: Supplementary file 2 — Additional file 2: The number of distribution age 95 to 105 for selected countries in 2010. (PDF 58 KB) [file 12877_2014_1048_MOESM2_ESM.pdf]

[Additional file 2] The number of distribution age 95 to 105 for selected countries in 2010

Age	Japan	Sweden	Canada	U.S.A	Spain	Italy	U.K.	Russia	Bulgaria	Norway	Ukraine
Female											
95	79516	4118	11796	96951	17048	31704	25222	18755	987	1790	6536
96	60592	3051	8520	71765	12493	23775	18704	12589	355	1292	4212
97	44420	2166	6116	52018	9225	17113	13159	8602	472	932	3047
98	31986	1399	4051	36143	6025	11104	8980	5184	308	614	1706
99	22008	873	2836	25000	3998	7639	6053	3365	239	381	1174
100	14631	582	1805	16403	2474	4661	3949	1975	117	213	641
101	9398	380	1148	10553	1525	2858	2519	1166	75	146	373
102	5717	197	701	6581	928	1578	1482	718	57	78	228
103	2904	102	419	3902	526	885	843	423	35	40	127
104	1791	55	235	2272	266	478	432	243	21	29	71
105	996	29	136	1295	132	252	219	143	11	14	38
Male											
95	20473	1292	3623	30121	5235	9593	7139	3012	491	524	1217
96	14305	877	2454	20321	3691	6770	4758	2069	172	317	809
97	9588	551	1639	13403	2546	4478	3104	1293	214	235	525
98	6321	327	988	8475	1634	2649	1935	773	150	130	309
99	4078	213	655	5338	1033	1720	1163	504	120	86	201
100	2473	118	372	3290	614	968	691	291	54	43	109
101	1469	68	213	1882	332	528	374	172	40	29	67
102	819	36	125	1133	204	260	190	110	27	12	38
103	433	14	67	607	122	132	99	65	16	8	21
104	231	7	32	320	57	64	45	37	9	5	11
105	105	0	14	160	24	28	23	23	5	3	6
