# Supplementary material for: Factors affecting the survival probability of becoming a centenarian for those aged 70, based on the human mortality database: income, health expenditure, telephone, and sanitation
Source: BMC Geriatr. 2014 Oct 21;14:113. doi: 10.1186/1471-2318-14-113 (PMC4216852; doi:10.1186/1471-2318-14-113)
Supplement: Supplementary file 3 — Additional file 3: A percentage distribution age 95 to 105 for selected countries in 2010. (PDF 58 KB) [file 12877_2014_1048_MOESM3_ESM.pdf]

[Additional file 3] A percentage distribution age 95 to 105 for selected countries in 2010

Age	Japan	Sweden	Canada	U.S.A	Spain	Italy	U.K.	Russia	Bulgaria	Norway	Ukraine
Female											
95	29.02	31.79	31.24	35.42	31.2	31.07	30.92	35.28	36.88	32.37	36.01
96	22.12	23.56	22.56	23.89	22.86	23.3	22.93	23.68	13.27	23.37	23.2
97	16.21	16.72	16.2	15.76	16.88	16.77	16.13	16.18	17.62	16.86	16.78
98	11.68	10.8	10.73	9.96	11.03	10.88	11.01	9.75	11.52	11.11	9.4
99	8.03	6.74	7.51	6.28	7.32	7.49	7.42	6.33	8.93	6.89	6.47
100	5.34	4.49	4.78	3.87	4.53	4.57	4.84	3.71	4.36	3.85	3.53
101	3.43	2.93	3.04	2.21	2.79	2.8	3.09	2.19	2.81	2.64	2.05
102	2.09	1.52	1.86	1.33	1.7	1.55	1.82	1.35	2.12	1.41	1.26
103	1.06	0.79	1.11	0.71	0.96	0.87	1.03	0.8	1.29	0.72	0.7
104	0.65	0.42	0.62	0.38	0.49	0.47	0.53	0.46	0.78	0.52	0.39
105	0.36	0.22	0.36	0.19	0.24	0.25	0.27	0.27	0.42	0.25	0.21
Male											
95	33.95	36.88	35.58	30.03	33.79	35.28	36.57	36.07	37.86	37.64	36.76
96	23.73	25.04	24.11	22.23	23.82	24.9	24.37	24.78	13.21	22.77	24.42
97	15.9	15.73	16.1	16.11	16.43	16.47	15.9	15.49	16.52	16.88	15.85
98	10.48	9.33	9.7	11.19	10.55	9.74	9.91	9.26	11.54	9.34	9.32
99	6.76	6.08	6.43	7.74	6.67	6.33	5.95	6.04	9.26	6.18	6.06
100	4.1	3.37	3.65	5.08	3.96	3.56	3.54	3.48	4.12	3.09	3.28
101	2.44	1.94	2.09	3.27	2.14	1.94	1.92	2.06	3.07	2.08	2.04
102	1.36	1.03	1.23	2.04	1.32	0.96	0.97	1.31	2.09	0.86	1.15
103	0.72	0.4	0.66	1.21	0.79	0.49	0.51	0.78	1.26	0.57	0.62
104	0.38	0.2	0.31	0.7	0.37	0.24	0.23	0.44	0.7	0.36	0.32
105	0.17	0	0.13	0.4	0.16	0.1	0.12	0.27	0.38	0.22	0.18
